# Tumor PD-L1 status and CD8^+^ tumor-infiltrating T cells: markers of improved prognosis in oropharyngeal cancer

**DOI:** 10.18632/oncotarget.19045

**Published:** 2017-07-06

**Authors:** Astrid De Meulenaere, Tijl Vermassen, Sandrine Aspeslagh, Philippe Deron, Fréderic Duprez, Debby Laukens, Jo Van Dorpe, Liesbeth Ferdinande, Sylvie Rottey

**Affiliations:** ^1^ Department of Medical Oncology, Ghent University Hospital, 9000 Ghent, Belgium; ^2^ DITEP, Gustave Roussy Cancer Centre, 94800 Villejuif, France; ^3^ Department of Head, Neck and Maxillo-Facial Surgery, Ghent University Hospital, 9000 Ghent, Belgium; ^4^ Department of Radiation Oncology, Ghent University Hospital, 9000 Ghent, Belgium; ^5^ Department of Internal Medicine, Ghent University Hospital, 9000 Ghent, Belgium; ^6^ Department of Pathology, Ghent University Hospital, 9000 Ghent, Belgium

**Keywords:** oropharynx, HPV, PD-L1, tumor infiltrating lymphocytes, biomarker

## Abstract

**Introduction:**

The aim of this study was to evaluate the expression of PD-L1 in oropharyngeal squamous cell carcinoma. Its relation with clinicopathological variables, tumor infiltrating lymphocytes and survival was also determined.

**Results:**

Positive PD-L1 status for the SP142 clone related with improved overall survival in oropharyngeal squamous cell carcinoma. Tumors heavily infiltrated by tumor infiltrating lymphocytes were also linked with better outcome, and this as well for the total number of tumor infiltrating lymphocytes as for the CD3^+^ and CD8^+^ T cell count. A Cox proportional hazard model proved that solely infiltrating CD8^+^ T cells exhibit a positive effect on overall survival (hazard ratio = 0.31 [0.14–0.70]; *P* = 0.0050)

**Materials and Methods:**

Formalin-fixed, paraffin-embedded tissue from oropharyngeal tumors of 99 patients was immunohistochemically stained for PD-L1 (SP142 and 22C3 clones), CD3, CD8 and FoxP3. Expression of PD-L1, CD3, CD8, FoxP3 and HPV status were correlated with clinicopathological variables. Overall survival was determined by a log-rank (Mantel–Cox) test whereas the Cox proportional hazard model was used for multivariate analysis.

**Conclusions:**

Our results demonstrate that CD8^+^ T lymphocytes constitute an independent prognostic marker in patients diagnosed with oropharyngeal squamous cell carcinoma. PD-L1 positivity for SP142, but not for 22C3, also tends to have a positive effect on survival in oropharyngeal squamous cell carcinoma.

## INTRODUCTION

Over the last decades there has been an increase in oropharyngeal squamous cell carcinoma (OSCC) which has mainly been attributed to human papillomavirus (HPV) infection [1, 2]. HPV-associated OSCC seems to be a distinct clinical entity, compared to tobacco related squamous cell carcinoma of the head and neck (SCCHN): patients (1) tend to be younger, (2) lack a history of smoking and/or alcohol abuse and (3) have a significant survival benefit [1, 3–9]. These observations have raised the possibility that some of these patients receive unnecessary treatment and are therefore exposed to unnecessary toxicity. Refining patient stratification by considering additional biomarkers next to HPV status may ensure appropriate therapy and therefore better survival and improved quality of life.

Programmed cell death protein 1 (PD-1) is a 50-55 kDa type I transmembrane receptor expressed by activated T and B cells, as well as by monocytes and dendritic cells. It has two binding partners, PD-L1 and PD-L2, each specific for various tissue types and with specific expression patterns. PD-L1 is expressed on immune cells (ICs) including T cells, dendritic cells and monocytes. In addition, PD-L1 is expressed in the context of T cell exhaustion during chronic viral infection (e.g. HPV infection) and by tumor cells (TCs) [10–12]. The presence of PD-L1 on TCs contributes to the process of immune evasion [13], hence PD-L1 expression is confirmed to be a marker of poor prognosis in the majority of solid tumors [14]. In the context of SCCHN (including OSCC) there is no consensus yet on the role of PD-L1 expression as a prognostic biomarker [15–18]. Multiple antibody (Ab) clones are currently analyzed for PD-L1 expression on cancer cells and in the cancer microenvironment using different immunohistochemical staining platforms or in-house staining procedures, often yielding different staining patterns. Moreover, read-out of PD-L1 positivity can be done on TCs as well as on ICs in the tumor microenvironment. This methodological complexity only contributes to the contradictorily results on the value of PD-L1 as biomarker. Therefore, the aim of this study was to perform additional research on the role of PD-L1 in SCCHN, focusing only on OSCC, using two clones from FDA approved kits, namely the 22C3 clone (Agilent-DAKO) and the SP142 clone (Roche), and scoring both TCs and ICs. We evaluated the expression of PD-L1 in OSCC and looked for associations with clinicopathological variables and the amount and type of tumor infiltrating lymphocytes (TILs). In addition we assessed the effect of PD-L1 status and the tumor immune cell infiltrate on outcome.

## RESULTS

### Patient characteristics

The 99 patients included in this study were predominantly male (83%), and represented primarily stage III/IV tumors (83%). Most of the tumors originated from the tonsil (43%) and were moderately (62%) differentiated. The overall HPV prevalence rate was 19% (19/99) with no difference in distribution along all topographic sites (*P* = 0.5673). More HPV^+^ specimens were detected among poorly differentiated and basaloid tumors (11/32) compared to well/moderately differentiated tumors (8/67; *P* = 0.0265).

Patients clinicopathological variables are displayed in Table 1.

**Table 1:** Association between PD-L1 on tumor cells, CD8^+^ T cells and clinicopathological variables

Parameter	Overall	SP142 PD-L1			22C3 PD-L1			CD8^+^ T cell count		
		Negative	Positive	*P*-value	Negative	Positive	*P*-value	Low	High	*P*-value
Patient number	99 (100)	72 (77)	22 (23)		64 (66)	33 (34)		67 (77)	20 (23)	
Age										
< 50 years	13 (13)	11 (11)	1 (1)	0.1522*	10 (10)	2 (2)	0.0234*	8 (9)	1 (1)	0.0248*
50–69 years	74 (75)	54 (57)	16 (17)		50 (52)	23 (24)		54 (62)	13 (15)	
≥ 70 years	12 (12)	7 (7)	5 (5)		4 (4)	8 (9)		5 (6)	6 (7)	
Gender										
Female	17 (17)	12 (13)	5 (5)	0.5351	12 (13)	5 (5)	0.7820	14 (16)	3 (3)	0.7515
Male	82 (83)	60 (64)	17 (18)		52 (54)	28 (29)		53 (61)	17 (20)	
Tumour site										
Tonsil	43 (43)	29 (31)	13 (14)	0.2108*	23 (24)	20 (21)	0.1621*	28 (32)	10 (12)	0.1330*
Tongue base	12 (12)	5 (5)	5 (5)		6 (6)	5 (5)		5 (6)	4 (5)	
Other sites	23 (23)	17 (18)	4 (4)		17 (18)	5 (5)		17 (20)	2 (2)	
Multiple subsites	21 (21)	21 (22)	0 (0)		18 (19)	3 (5)	–	17 (20)	4 (5)	–
T stage										
T_1-2_	56 (57)	39 (42)	16 (17)	0.1441	32 (33)	23 (24)	0.0650*	32 (37)	17 (20)	0.0041
T_3-4_	43 (43)	33 (35)	6 (6)		32 (33)	10 (10)		35 (40)	3 (3)	
N stage										
N_0_	30 (30)	23 (25)	4 (4)	0.2100*	21 (22)	8 (8)	0.0939*	19 (22)	5 (6)	0.6038*
N_1_	14 (14)	12 (13)	2 (2)		12 (12)	2 (2)		12 (14)	2 (2)	
N_2-3_	55 (56)	37 (39)	16 (17)		31 (32)	23 (24)		36 (41)	13 (15)	
Prognostic stage										
I/II	17 (17)	15 (16)	2 (2)	0.3430	12 (13)	5 (5)	0.7820	10 (12)	4 (5)	0.7291
III/IV	82 (83)	57 (61)	20 (21)		52 (54)	28 (29)		57 (66)	16 (18)	
Grade of differentiation										
Well/moderately	67 (68)	58 (62)	7 (7)	0.0001*	50 (52)	16 (17)	0.0111*	52 (60)	8 (9)	0.0006*
Poorly	19 (19)	8 (8)	10 (10)		8 (8)	11 (11)		6 (7)	9 (10)	
Basaloid	13 (13)	6 (6)	5 (5)		6 (6)	6 (6)		9 (10)	3 (3)	
HPV status										
Negative	80 (81)	66 (70)	11 (12)	**< 0.0001**	55 (57)	23 (24)	0.0649	57 (66)	13 (15)	0.0591
Positive	19 (19)	6 (6)	11 (12)		9 (9)	10 (10)		10 (12)	7 (8)	
Primary therapy										
Surgery	51 (52)	34 (36)	15 (16)	0.1374*	31 (31)	19 (20)	0.4390*	32 (37)	11 (13)	0.0521*
Radiotherapy	25 (25)	18 (19)	5 (5)		16 (17)	9 (9)		15 (17)	8 (9)	
Chemoradiotherapy	23 (23)	20 (21)	2 (2)		17 (18)	5 (5)		20 (23)	1 (1)	

Data given are numbers, percentages are given between brackets (%). Overall column displays total numbers (%). Cut-off values were taken at median values (3+: abundant occurrence of CD8^+^ T cells) and at ≥ 5% for PD-L1 on TCs (SP142 clone and 22C3 clone). Samples that were classified as ‘impossible to evaluate’ were not included in the analysis. *P*-value calculated by Fisher exact test unless otherwise indicated (*P-value calculated by Chi-square test). HPV, human papillomavirus.

### The pattern of PD-L1 expression

#### Tumor cells (TCs)

Using the SP142 clone for evaluation of PD-L1 expression, PD-L1 status of TCs was categorized as follows: IHC 0 (58%), IHC 1 (15%), IHC 2 (12%) and IHC 3 (10%). Five samples (5%) could not be evaluated. Using the 22C3 clone, distribution of tumoral IHC scores was as follows: IHC 0 (45%), IHC 1 (20%), IHC 2 (13%) and IHC 3 (20%). Two samples (2%) could not be evaluated. PD-L1 positivity defined as ≥ 5% PD-L1 expression on TCs resulted in 23% (22/94) and 34% (33/97) PD-L1^+^ tumor specimens for the SP142 and 22C3 clone, respectively (Table 1).

#### Immune cells (ICs)

Only little tumor infiltrating ICs expressed PD-L1 (SP142 clone) with IHC 0 in 76 cases (77%), IHC 1 in 9 cases (9%), IHC 2 in one case (1%) and IHC 3 in one case (1%). Twelve samples (12%) were not evaluable. Similar to the SP142 clone, low expression was observed on ICs for the 22C3 clone: IHC 0 in 66% of cases, IHC 1 in 16% of cases, IHC 2 in 3% of cases and no IHC 3. Thirteen samples could not be evaluated.

### Relation of PD-L1 expression on TCs with different clinicopathological variables

A positive link was found between HPV status and PD-L1 positivity on TCs for the SP142 clone (*P <* 0.0001) and 22C3 clone (*P* = 0.0649). Next, positive SP142 PD-L1 expression was associated with poorly differentiated/basaloid tumors (15/29 versus 7/65; *P* = 0.0001) whereas positive 22C3 PD-L1 expression on TCs was most common in elder patients (8/12 versus 25/85; *P* = 0.0234) and in poorly differentiated/basaloid tumors (17/31 versus 16/66; *P* = 0.0111).

All associations between PD-L1 on TCs and clinicopathological variables are presented in Table 1.

### Relation of PD-L1 expression on TCs with TILs

Notably, PD-L1 positivity on TCs was linked with the immune environment, as this parameter was associated with increased TIL count (*P <* 0.0001 and *P* = 0.0009), CD3^+^ T cell count (*P* = 0.0009 and *P* = 0.0044), CD8^+^ T cell count (*P <* 0.0001 and *P* = 0.0001) and FoxP3^+^ T cell count (*P* = 0.0004 and *P* = 0.0094) for the SP142 and 22C3 clone, respectively.

All associations with TILs are given in Table 2.

**Table 2:** Association between PD-L1 on tumor cells and stromal immune cell infiltrate

Parameter	Overall	SP142 PD-L1			22C3 PD-L1		
		Negative	Positive	*P*-value	Negative	Positive	*P*-value
Patient number	99 (100)	72 (77)	22 (23)		64 (66)	33 (34)	
TIL count							
1+	12 (12)	12 (14)	0 (0)	**< 0.0001**	11 (12)	1 (1)	**0.0009**
2+	42 (42)	37 (42)	3 (3)		32 (36)	9 (10)	
**3+**	26 (26)	14 (16)	11 (13)		13 (14)	13 (14)	
4+	11 (11)	4 (5)	7 (8)		3 (3)	8 (9)	
ITE	8 (8)						
CD3^+^ T cell count							
1+	20 (20)	18 (21)	0 (0)	**0.0009**	15 (17)	4 (5)	**0.0044**
2+	33 (33)	28 (32)	5 (6)		26 (29)	8 (9)	
**3+**	19 (19)	12 (14)	7 (8)		12 (14)	7 (8)	
4+	17 (17)	8 (9)	9 (10)		5 (6)	12 (14)	
ITE	10 (10)						
CD8^+^ T cell count							
1+	36 (36)	34 (40)	2	**< 0.0001**	30 (35)	7 (8)	**0.0001**
2+	31 (31)	24 (28)	5 (6)		22 (25)	8 (9)	
**3+**	20 (20)	8 (9)	12 (14)		5 (6)	15 (17)	
ITE	12 (12)						
FoxP3^+^ T cell count							
1+	58 (59)	51 (59)	7 (8)	0.0004*	44 (49)	15 (17)	**0.0094**
**2+**	31 (31)	15 (17)	14 (16)		14 (16)	16 (18)	
ITE	10 (10)						

Data given are numbers, percentages are given between brackets (%). Cut-off values were taken at ≥ 5% for PD-L1 on TCs (SP142 clone and 22C3 clone). Parameters in bold and underlined represent median cut-off values used for survival analysis: 3+ (TIL count, CD3^+^ T cell count and CD8^+^ T cell count) and 2+ (FoxP3^+^ T cell count). Samples that were classified as ‘impossible to evaluate’ were not included in the analysis. *P*-value calculated by Chi-square test unless otherwise indicated (* P-value calculated by Fisher exact test). ITE, impossible to evaluate; TIL, tumor-infiltrating lymphocyte.

### Relation of PD-L1 expression on TCs with clinical outcome

**Figure 1:**
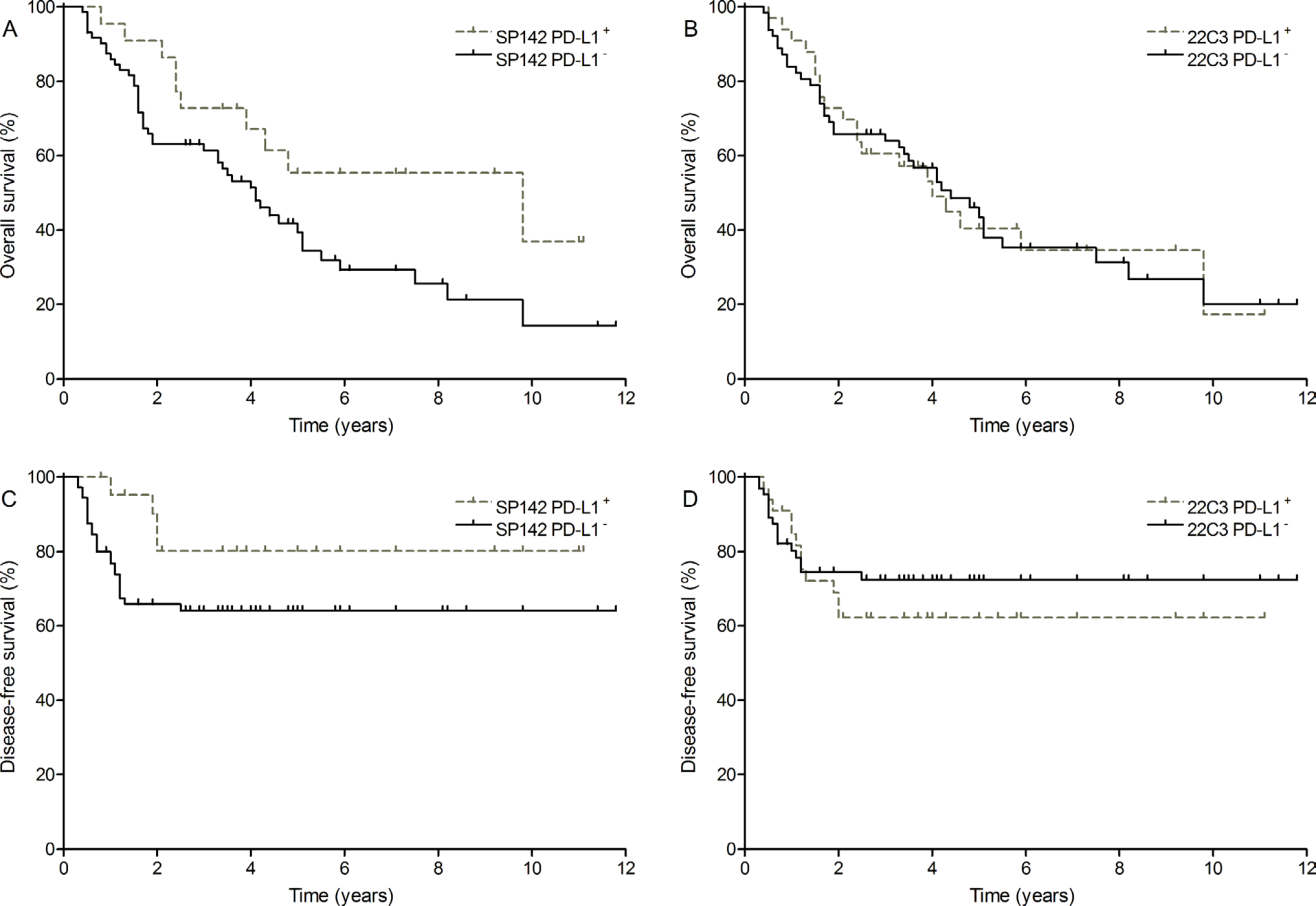
Survival outcome of PD-L1 expression (**A**) OS of SP142 PD-L1 (0.51 [0.31–0.99], *P* = 0.0466); (**B**) OS of 22C3 PD-L1 (0.99 [0.58–1.72], *P* = 0.9846); (**C**) DFS of SP142 PD-L1 (0.43 [0.22– 1.16], *P* = 0.1070) and (**D**) DFS of 22C3 PD-L1 (1.28 [0.60–2.83], *P* = 0.5110). Cut-off values were taken at ≥ 5% for PD-L1 on TCs (SP142 and 22C3 clone).

Positive PD-L1 expression using the SP142 clone resulted in a more favorable outcome with median overall survival (OS) of 9.8 years versus 4.1 years for SP142 PD-L1 negativity (hazard ratio [HR] = 0.51 [0.31–0.99], *P* = 0.0466; Figure 1A) whereas 22C3 PD-L1 expression on TCs showed no significant association with OS (4.0 years versus 4.4 years; HR = 0.99 [0.58–1.72], *P* = 0.9846; Figure 1B). None of the PD-L1 markers was associated with prolonged disease-free survival (DFS) (HR SP142 = 0.43 [0.22–1.16], *P* = 0.1070 and HR 22C3 = 1.28 [0.60–2.83], *P* = 0.5110; Figure 1C–1D). An overview of the HRs on DFS for all tumor markers, IC markers and associated clinicopathological markers is given in Supplementary Table 1.

**Figure 2:**
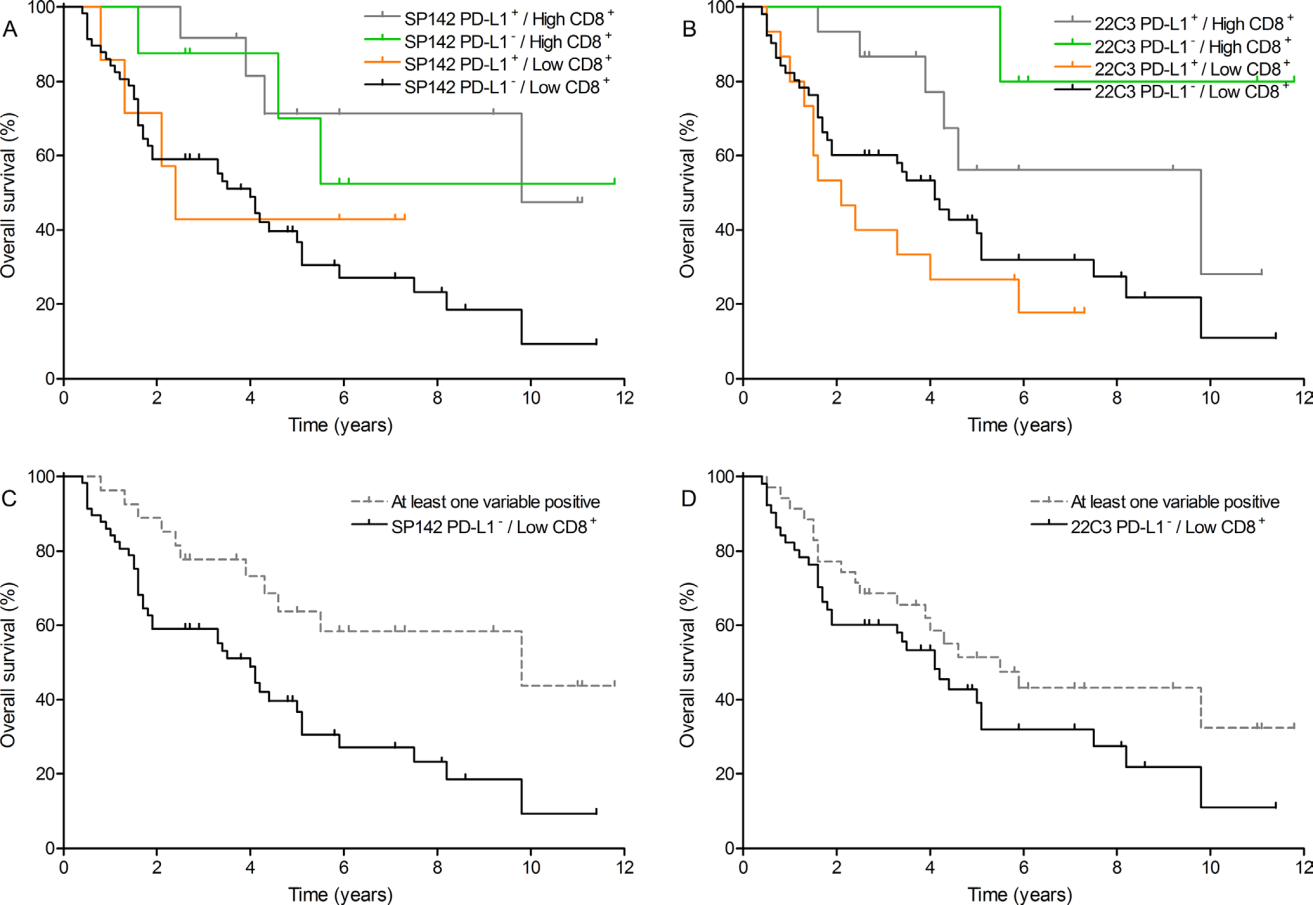
Overall survival outcome combining PD-L1 expression on tumor cells with CD8^+^ T cell count (**A**) SP142 PD-L1/CD8^+^ T cell count: all groups (*P* = 0.0321); (**B**) 22C3 PD-L1/CD8^+^ T cell count: all groups (*P* = 0.0118); (**C**) SP142 PD-L1/CD8^+^ T cell count: PD-L1^−^/low CD8^+^ versus at least one variable positive (0.40 [0.24–0.78], *P* = 0.0048) and (**D**) 22C3 PD-L1/CD8^+^ T cell count: PD-L1^−^/low CD8^+^ versus at least one variable positive (0.66 [0.38–1.15], *P* = 0.1415). Cut-off values were taken at median values (3: abundant occurrence of CD8+ T cells) and at ≥ 5% for PD-L1 on tumor cells (SP142 and 22C3 clone).

The relation between TILs and outcome was evaluated as well. High TIL count, high CD3^+^ T cell count and high CD8^+^ T cell count but not FoxP3^+^ T cell count proved to have a favorable influence on OS (Supplementary Figure 1). Particularly, an elevated CD8^+^ T cell count was significantly associated with prolonged OS (9.8 years) versus patients with a low CD8^+^ T cell count (3.5 years; HR = 0.32 [0.22–0.71], *P* = 0.0020). As CD8^+^ T cell count proved the strongest marker of the immune cell infiltrate, an analysis was performed combining CD8^+^ T cell count and PD-L1 expression on TCs. The combined analysis of SP142 PD-L1 expression on TCs and CD8^+^ T cell count showed that this combination was significantly correlated with OS: median OS of 9.8 years, not reached, 2.4 years and 4.0 years for the PD-L1^+^/high CD8^+^, PD-L1^−^/high CD8^+^, PD-L1^+^/low CD8^+^, and PD-L1^−^/low CD8^+^ cohorts, respectively (*P* = 0.0321; Figure 2A). Similar, a significance was found for combining 22C3 PD-L1 expression on TCs and CD8^+^ T cell count with median OS of 9.8 years, not reached, 2.1 years and 4.1 years for the respective cohorts (*P* = 0.0118; Figure 2B). As the majority of patients were stratified as PD-L1^−^/low CD8^+^, patients were bicategorically categorized as ‘at least one variable positive’ versus PD-L1^−^/low CD8^+^. Patients with ‘at least one variable positive’ had a more favorable outcome for the SP142 PD-L1 expression (median OS 9.8 years versus 4.0 years; HR = 0.40 [0.24–0.78], *P* = 0.0048; Figure 2C) but not for the 22C3 PD-L1 expression (median OS 5.5 years versus 4.1 years; HR = 0.66 [0.38–1.15], *P* = 0.1415; Figure 2D).

In the multivariate model, which adjusted for HPV status, CD8^+^ T cell count proved to be the independent prognosticator for OS (HR = 0.31 [0.14–0.70]; *P* = 0.0050) whereas SP142 PD-L1 positivity and other immune cell counts did not contribute to the model. When assessing the combination of SP142 PD-L1 expression and CD8^+^ T cell count in the multivariate model, patients with ‘at least one variable positive’ had an OS benefit compared with PD-L1^−^/low CD8^+^ patients (HR = 0.39 [0.20–0.78]; *P* = 0.0070).

An overview of all multivariate HR is given in Table 3.

**Table 3:** Multivariate Cox regression model for overall survival outcome

Parameter	Separate immune markers		Combination of immune markers	
	Multivariate HR (95% CI)	*P*-value	Multivariate HR (95% CI)	*P*-value
HPV status				
Negative	1		1	
Positive	0.55 (0.21–1.43)	0.2213	0.61 (0.25–1.49)	0.2786
TIL count				
Low	1		1	
High	1.06 (0.48–2.35)	0.8828	1.11 (0.49–2.52)	0.7976
CD3^+^ T cell count				
Low	1		1	
High	0.94 (0.45–1.97)	0.8660	0.77 (0.37–1.61)	0.4859
CD8^+^ T cell count				
Low	1			
High	**0.31 (0.14–0.70)**	**0.0050**		
SP142 PD-L1				
Negative	1			
Positive	0.98 (0.35–2.74)	0.9689		
Combination SP142 PD-L1/CD8^+^ T cell count				
PD-L1^−^/low CD8 ^+^			1	
At least 1 positive/high			**0.39 (0.20–0.78)**	**0.0070**

Multivariate HRs were calculated through a Cox proportional-hazards regression model. Multivariate analysis was performed for CD8^+^ T cell count and SP142 PD-L1 as separate immune markers or as combined immune markers. Cut-off values were taken at median values (3+: abundant occurrence of TILs, CD3^+^ T cells and CD8^+^ T cells) and at ≥ 5% for PD-L1 on tumor cells (SP142 clone). HPV, human papillomavirus; HR, hazard ratio; TIL, tumor-infiltrating lymphocyte.

### Relation of PD-L1 expression on ICs with clinicopathological variables, TILs and clinical outcome

No relation was demonstrated between PD-L1 expression (SP142 or 22C3 clone) on ICs and clinicopathological variables, including TILs, overall and disease-free survival.

## DISCUSSION

In our study, positive PD-L1 expression on TCs using the SP142 clone was noticed in 23% of specimens whereas 34% of specimens were PD-L1^+^ according to the 22C3 clone (≥ 5 % cut-off). The number of PD-L1^+^ samples is considerably lower in comparison to other previously reported data on the expression of PD-L1 in OSCC [19, 20]. These differences might be partially explained by the difference in sample size. However, the main confounder for the lower PD-L1 expression is probably the use of different Ab clones. Each Ab targets different epitopes of PD-L1 with different affinity, resulting in different staining patterns [21]. When considering PD-L1 as a biomarker in daily clinical practice, one should be aware of these methodological concerns and validation and quality assessment of this immunohistochemical marker will prove to be of the utmost importance.

The study of PD-L1 expression on ICs did not add useful data to our study, probably because of the very low expression levels (98% of the samples using the SP142 clone and 97% of the samples using the 22C3 clone [≥ 5% cut-off] were PD-L1^−^). The expression of PD-L1 on tumor infiltrating ICs is well known but further research is warranted in order to evaluate its contribution to the (anti-) tumor immune response and its link with PD-L1 expression on TCs.

We found PD-L1 expression on TCs determined by SP142 and 22C3 staining to be positively associated with high number of TILs, high CD3^+^ T cell count, high CD8^+^ T cell count and high FoxP3^+^ T cell count. These findings reflect the hypothesis that a PD-L1 mediated adaptive immune resistance is induced following interferon-gamma secretion by TILs [22]. It is reported that PD-L1 expression is visually higher in areas with high T cell infiltrate and this finding is confirmed in our study [23]. PD-L1 was also associated with positive HPV status which in turn was nearly significantly associated with CD8^+^ T cells. As PD-L1 expression is observed in the deep invaginations of tonsillar crypts in non-cancerous tonsils as well, this observation might suggest that virus induced malignant transformation could be enhanced in the reticulated epithelium of tonsillar crypts in the presence of PD-L1.

Survival analysis revealed that PD-L1 positivity of TCs, using the SP142 clone, was related to better OS but not with DFS in univariate analysis. Also high CD8^+^ T cell count showed prognostic OS benefit in OSCC, irrespective of the HPV status. As both CD8^+^ T cell count and PD-L1 status were related to a better OS, their effect was even more pronounced when using a combined prognostic biomarker based on PD-L1 status plus CD8^+^ T cell count. Having ‘at least one variable positive’ resulted in an increased survival outcome compared to patients who were stratified as PD-L1^−^/low CD8^+^. The link of PD-L1 with T-cell infiltrate and HPV status as demonstrated by our own study should at least partially be accounted for this observation. The fact that PD-L1^+^ tumors and high infiltration of CD8^+^ T lymphocytes showed better survival outcome might come across as a paradox since high CD8^+^ lymphocyte count indicates an active immune response with anti-tumor effect whereas PD-L1 positivity is assumed to dampen this response. This observation demonstrates that PD-L1 expression cannot be interpreted simply as a marker of immune suppression but rather reflects a response to (over-) activation of an endogenous inflammatory immune response at the tumor site [23, 24].

To our knowledge, this is the first study to evaluate the expression of PD-L1 in OSCC using two monoclonal Abs (SP142 clone and 22C3 clone) from FDA approved kits that are currently used in various clinical trials. Pharmacodiagnostic testing for anti-PD-(L)1 therapy is nowadays mainly performed with two clones from Agilent-DAKO and two clones from Roche. Recently, it was reported that staining results for the 22C3 clone are in concordance with those obtained from the 28-8 and SP263 clone, but not with those of the SP142 clone [25, 26] and this incited us to focus on the 22C3 and SP142 clone for this study. Besides, we chose to include tumors arising from one primary tumor site. Although SCCHN is often considered one entity, recent publications report a heterogeneous molecular and immunological tumor profile of these malignancies on different anatomical localizations [27, 28]. Therefore it is desirable to focus on one tumor site rather than on SCCHN in general and thus we restricted the assessment of PD-L1 expression to a well-defined population of patients with tumors arising from the oropharynx.

Also, we deliberately chose to work with formalin-fixed, paraffin-embedded (FFPE) samples from biopsies or resections as this is the material that will be available for prognostic biomarker testing in the future. Although biopsy samples might harbor sampling errors and suffer from spatial heterogeneity in PD-L1 expression, it is important to include these samples in the trials to avoid selection bias. It remains however important to evaluate the concordance of PD-L1 expression between different available clones, between different cut-off values and between samples from different origins (e.g. biopsy versus resection specimens).

In conclusion our results demonstrate that CD8^+^ TILs constitute a strong positive prognostic marker in patients diagnosed with OSCC. Furthermore, PD-L1 expression on TCs, assessed by the SP142 clone, also tends to have a positive effect on patient outcome in OSCC. The results of this retrospective study should be confirmed in prospective trials to assess the clinical impact of the biomarkers on routine daily practice. Herein, special attention should be given to methodological issues on PD-L1 testing that were also of concern in this study, such as choice of Ab clone, interpretation criteria and sample type.

## MATERIALS AND METHODS

### Study population

Ninety-nine patients with histologically proven OSCC (anno 2004-2013) were selected from the archival database of the Department of Pathology, Ghent University Hospital. Patients were excluded from the study in case of recurrent or distant metastatic disease. When available, samples from resection specimens were preferred for further analysis (*n* = 27), if not, endoscopic biopsy material was used (*n* = 72).

The study was approved by the ethics committee of the Ghent University Hospital.

Clinical, treatment and follow-up data were retrieved from the electronic patient data file. Tumor sites consisted of tonsil, tongue base, other (e.g. tonsillar pillars, posterior wall, vallecula) or multiple subsites involved. The majority of the patients was treated surgically (*n* = 51) by either tumor resection with or without lymph node dissection (*n* = 19 and *n* = 8, respectively) or lymph node dissection alone (*n* = 24). Forty-six out of 51 surgically patients received adjuvant treatment, that is, concurrent chemoradiotherapy (*n* = 30) or radiotherapy alone (*n* = 16). Clinical data and treatment details are presented in Table 1. For all patients, treatment decisions were discussed in a multidisciplinary meeting consisting of dedicated head and neck surgeons, medical and radiation oncologists, radiologists and pathologists.

### HPV status

HPV status was determined on FFPE material by high-risk HPV *in situ* hybridization (ISH) using the Inform HPV III Family B probe (Ventana Medical Systems) on a BenchMark XT automated stainer (Ventana Medical Systems) under ISO15189:2012 accreditation. The probe cocktail detects HPV types 16, 18, 31, 33, 35, 39, 45, 51, 52, 56, 58 and 66. The high-risk HPV ISH test was considered positive if a discrete, blue colored, precipitated reaction product within the TCs was observed.

### Immunohistochemistry

Immunohistochemistry for PD-L1 and TILs subtyping was performed using the BenchMark XT instrument (Ventana Medical Systems inc., Arizona, USA). Staining of CD3, CD8 and FoxP3 (DAKO, Glostrup, Denmark) was performed on FFPE tissue slides of 2μm as previously described [29]. For staining of PD-L1, similar antigen retrieval and blocking steps were followed by incubation with the anti-PD-L1 primary Ab (clone 22C3 diluted 1:100 [Agilent-DAKO, United States] and clone SP142 ready-to-use [Roche, Basel, Switzerland]) and DAB detection using the Ultraview kit (Ventana) and the optiview kit (Ventana), respectively, according to the instructions of the manufacturer. Slides were counterstained with hematoxylin.

### Scoring of the tumor samples

First, a hematoxylin-eosin slide was evaluated to confirm the presence of invasive squamous cell carcinoma, to determine the grade of tumor differentiation and/or define basaloid subtype. The infiltration of TILs in tumor stroma was scored semi-quantitatively with a 4-tiered scale (1+ to 4+) as previously described [29, 30]. Representative scoring for stromal TILs is given in Supplementary Figure 2.

Elaborate evaluation of the immune infiltrate in the stromal compartment (i.e. lymphocytes within the intertumoral stroma), was done semi-quantitatively by scoring for CD3^+^, CD8^+^ and FoxP3^+^ T cell count. For the subtyping of the immune infiltrate, three representative fields were selected and scored using a similar 4-tiered semi-quantitative method as for TIL scoring (1+ to 4+) [29, 30] (Supplementary Figure 2). The mean of the separate scores from each of the 3 fields was calculated as final score, ranging from 1+ to 4+. Necrotic or ulcerated areas were excluded from evaluation. Criteria to classify samples as ‘impossible to evaluate (ITE)’ were the following: 1) (virtually) no tumor stroma present, 2) presence of only a minimal invasive carcinoma component and 3) the inability to differentiate between the ICs of the pre-existing lymphoid tissue of the tonsil and the tumor-associated IC infiltrate.

**Figure 3:**
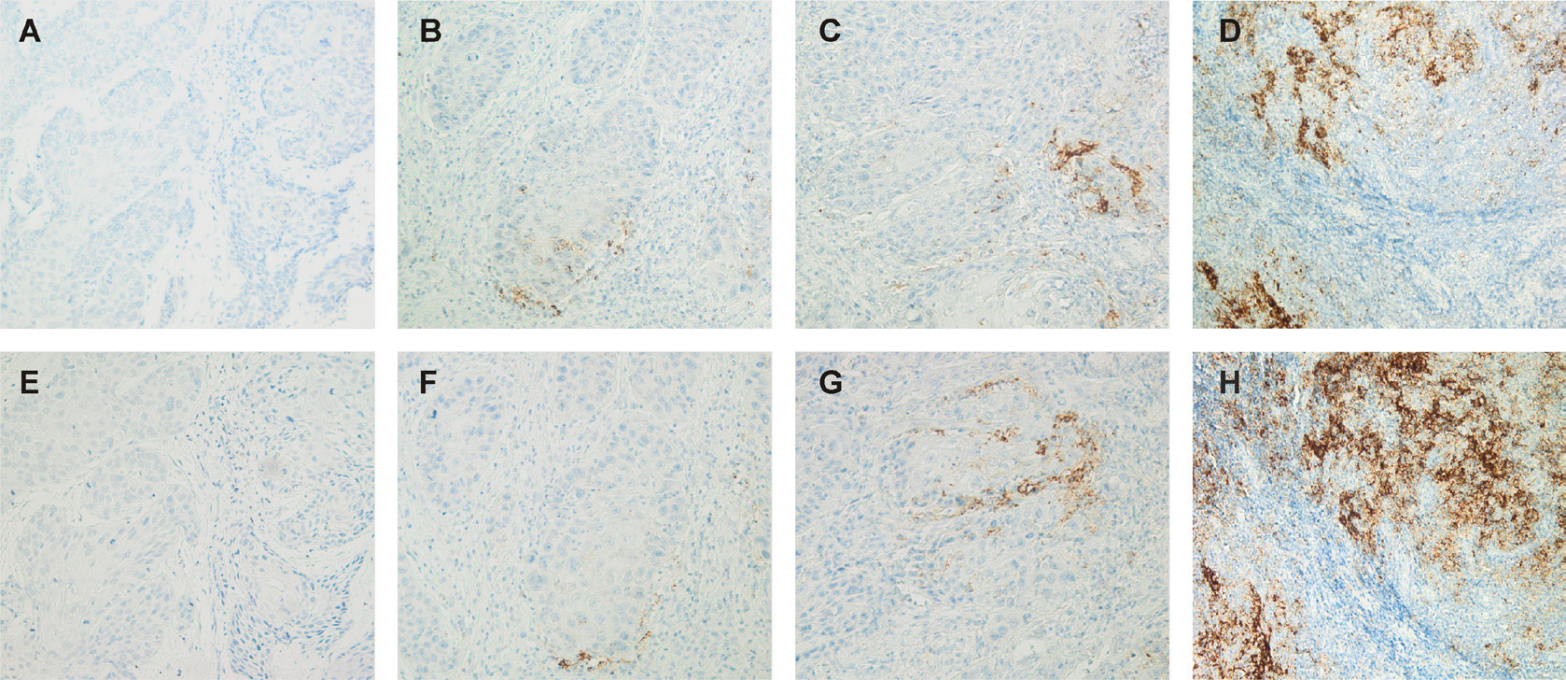
Representative immunohistochemical staining for PD-L1 (x200) PD-L1 expression was determined on TCs. Staining patterns for SP142 PD-L1 were: IHC 0 (**A**), IHC 1 (**B**), IHC 2 (**C**) and IHC 3 (**D**). Staining patterns for 22C3 PD-L1 were: IHC 0 (**E**), IHC 1 (**F**), IHC 2 (**G**) and IHC 3 (**H**). Staining with SP142 and 22C3 clone was performed on serial sections (2 μm) of the same tumor sample for each IHC score respectively.

Subsequently, samples were scored for PD-L1 expression with both the SP142 clone and the 22C3 clone by two independent observers. IHC was scored 0 if < 1% of TCs were positive, IHC 1 if ≥ 1% but < 5% of TCs were positive, IHC 2 if ≥ 5% but < 10% of TCs were positive or IHC 3 if ≥ 10% of TCs were positive (Figure 3) [31]. Mean scores between both observers were used in the analyses. Cut-off values for PD-L1 positivity on TCs were taken at ≥ 5% (membranous and/or cytoplasmic) for the SP142 and 22C3 clone. PD-L1 expression on ICs was evaluated using Cell^^^D imaging software (Olympus Corporation, Tokyo, Japan) to ensure objective scoring as the expression of PD-L1 on ICs was low. PD-L1 staining was quantified in the three same representative fields of intertumoral stroma used for scoring of the T cell infiltrate. Scores were given as percentages reflecting the ratio of PD-L1 staining surface area to the whole surface area of the representative field. The final score was determined as the mean of the separate scores from the chosen fields. Again, cut-off values for PD-L1 positivity on ICs were taken at ≥ 5%.

### Statistical analysis

Sample size calculation for a proportional *z* test of the most fitting immune cell marker (CD8^+^ T cells) has shown that 106 patients are needed to achieve a power of 90% (α = 5%; proportion event group high CD8^+^ = 0.35; proportion event low CD8^+^ = 0.68; sample ratio high CD8^+^/ low CD8^+^ = 0.29). Associations between categorical variables were determined via Chi-square test. In case of 2 × 2 contingency tables with 1 or more observed values lower than 10, a Fisher's Exact test was applied. Secondly, the HR of immunohistochemical parameters on OS and DFS was determined by a log-rank (Mantel–Cox) test. OS was calculated from time from diagnosis on biopsy until day of death or final follow-up whereas DFS was calculated from time of therapy until time of recurrence. For every patient who was not seen in clinic for the preceding 12 months at the point of the survival analysis, the general practitioner was contacted to confirm whether the patient was still alive. Patients that were lost to follow up at either the Ghent University Hospital or the general practitioner were censored in the survival analysis (*n* = 8). Finally, the covariate effect of the risk factors on survival analysis, that reached a *P* value lower than 0.1 on univariate log-rank test, was determined by means of the Cox proportional hazard model (backward method). Samples that were classified as ‘ITE’ were not included in the analysis.

## SUPPLEMENTARY MATERIALS FIGURES AND TABLE


