# Supplementary material for: Tumor PD-L1 status and CD8+ tumor-infiltrating T cells: markers of improved prognosis in oropharyngeal cancer
Source: Oncotarget. 2017 Jul 6;8(46):80443–52. doi: 10.18632/oncotarget.19045 (PMC5655210; doi:10.18632/oncotarget.19045)
Supplement: Supplementary file 1 [file oncotarget-08-80443-s001.pdf]

Tumor PD-L1 status and CD8+ tumor-infiltrating T cells: markers of improved prognosis in oropharyngeal cancer

Supplementary Materials

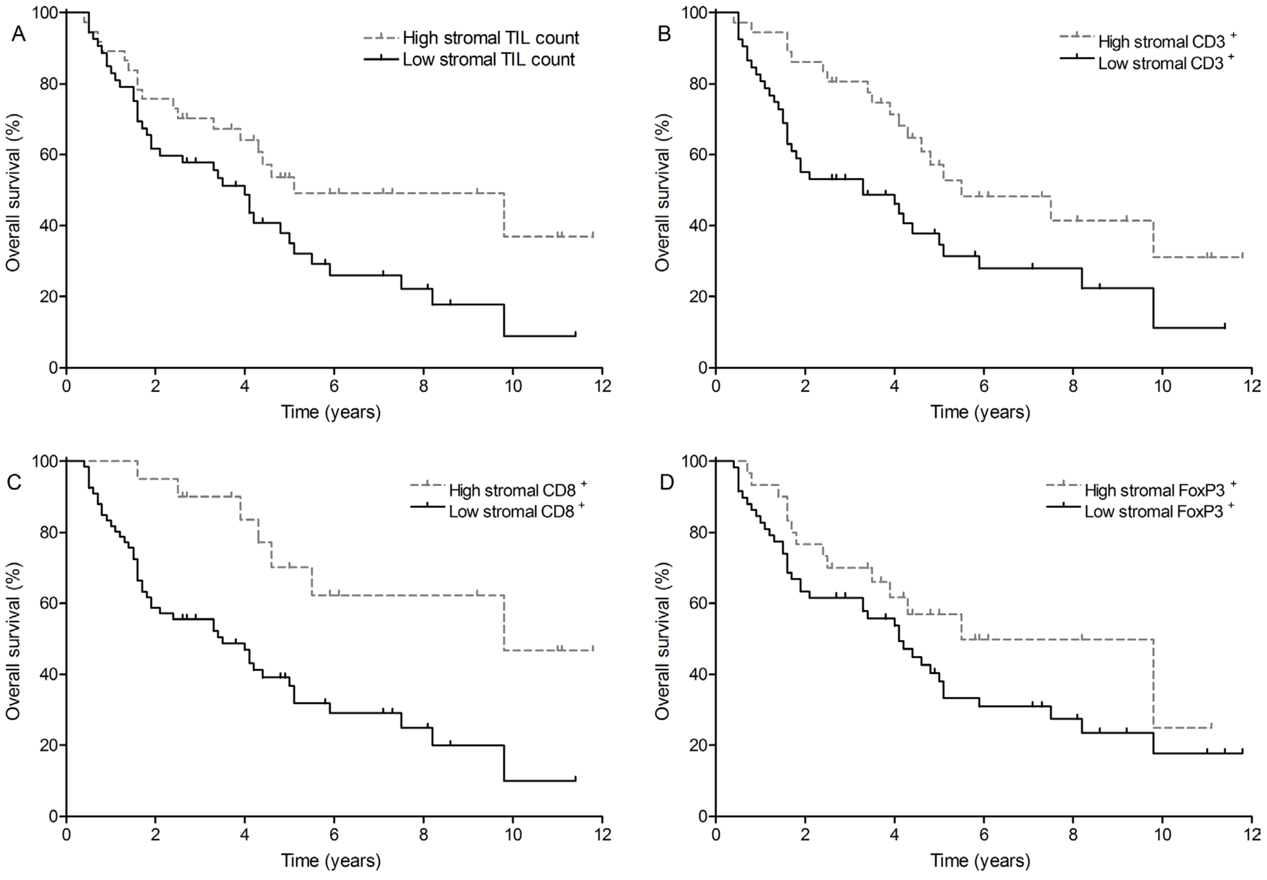

Supplementary Figure 1: Overall survival outcome of stromal immune cell infiltrate. (**A**) TIL count (0.55 [0.33–0.96], P = 0.0335); (**B**) CD3+ T cell count (0.50 [0.29–0.87], P = 0.0139); (**C**) CD8+ T cell count (0.32 [0.22–0.71], P = 0.0020) and (**D**) FoxP3+ T cell count (0.63 [0.37–1.14], P = 0.1292). Cut-off values (low versus high) were taken at median values: 3+ (TIL count, CD3+ T cell count and CD8+ T cell count) and 2+ (FoxP3+ T cell count). TIL, tumor-infiltrating lymphocyte.

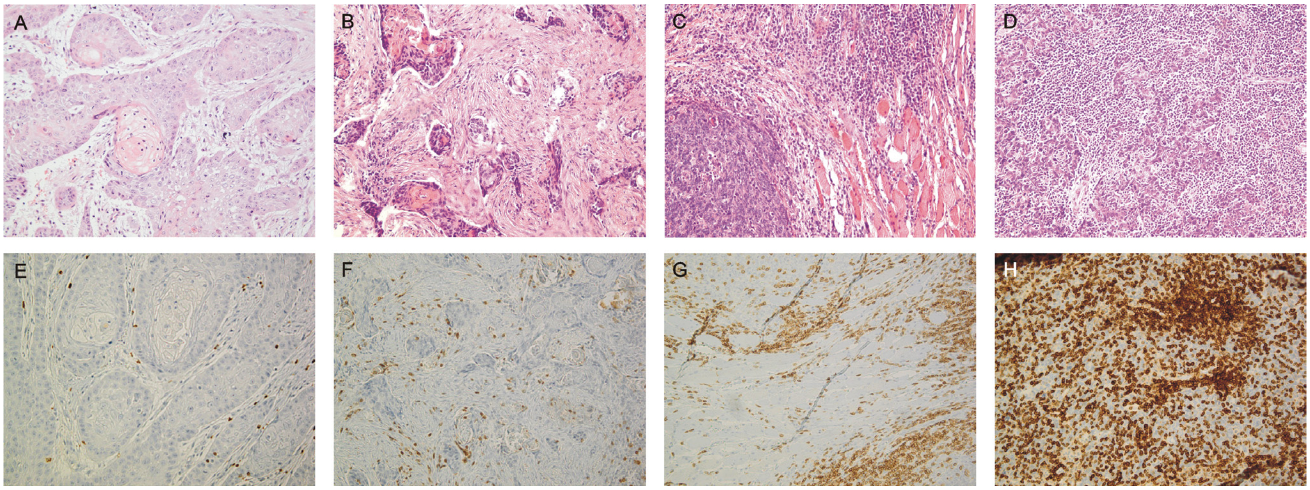

Supplementary Figure 2: Representative hematoxylin-eosin staining for presence of tumor infiltrating lymphocytes and CD3$^+$ T cell count (x200). TIL count was scored semi-quantitatively as follows: (**A**) 1$^+$, (**B**) 2$^+$, (**C**) 3$^+$ and (**D**) 4$^+$. CD3$^+$ T cell count was scored semi-quantitatively as follows: (**E**) 1$^+$, (**F**) 2$^+$, (**G**) 3$^+$ and (**H**) 4$^+$.

Supplementary Table 1: Disease-free survival

Clinicopathological variables		
**Parameter**	**Univariate HR (95% CI)**	***P*-value**
Age		
< 50 years	1	
50 – 69 years	0.65 (0.23–1.83)	0.3338
≥ 70 years	0.32 (0.08–1.28)	0.1368
T stage		
T1-2	1	
T3-4	1.58 (0.75–3.32)	0.2062
N stage		
N0	1	
N1	0.43 (0.12–1.49)	0.2534
N2-3	1.10 (0.50–2.42)	0.8096
Grade of differentiation		
Well / moderately	1	
Poorly	0.72 (0.30–1.73)	0.4939
Basaloid	0.45 (0.15–1.30)	0.2576
Primary treatment		
Surgery	1	
Radiotherapy	0.65 (0.26–1.71)	0.4001
Chemoradiotherapy	1.41 (0.60–3.55)	0.4036
**Stromal immune cell infiltrate**		
Parameter	Univariate HR (95% CI)	*P*-value
TIL count		
Low	1	
High	0.70 (0.32–1.50)	0.3548
$CD3^+$ T cell count		
Low	1	
High	0.82 (0.37–1.81)	0.6107
$CD8^+$ T cell count		
Low	1	
High	0.51 (0.23–1.38)	0.2050
$FoxP3^+$ T cell count		
Low	1	
High	0.65 (0.29–1.51)	0.3280
**Tumor**		
Parameter	Univariate HR (95% CI)	P-value
HPV status		
Negative	1	
Positive	0.39 (0.20–1.16)	0.1033
SP142 PD-L1		
Low	1	
High	0.43 (0.22–1.16)	0.1070
22C3 PD-L1		
Low	1	
High	1.28 (0.60–2.83)	0.5110
Combination SP142 PD-L1 / $CD8^+$ T cell count		
$PD\text{-}L1^-$ / low $CD8^+$	1	
At least 1 high	0.44 (0.21–1.11)	0.0849
Combination 22C3 PD-L1 / $CD8^+$ T cell count		
$PD\text{-}L1^-$ / low $CD8^+$	1	
At least 1 high	1.04 (0.46–2.36)	0.9167

Univariate HR was calculated via Kaplan-Meier survival analysis and log-rank test. Cut-off values were taken at median values (3^+: abundant occurrence of TILs, $CD3^+$ T cells and $CD8^+$ T cells; and 2^+: moderate numbers of $FoxP3^+$ T cells) and at ≥ 5% for PD-L1 on TCs (SP142 clone and 22C3 clone). Samples that were classified as 'impossible to evaluate' were not included in the survival analysis. HPV, human papillomavirus; HR, hazard ratio; TIL, tumor-infiltrating lymphocyte.
